# Comparison of the Effects of Acute and Chronic Administration of Tetrahydroisoquinoline Amines on the In Vivo Dopamine Release: A Microdialysis Study in the Rat Striatum

**DOI:** 10.1007/s12640-016-9661-1

**Published:** 2016-08-27

**Authors:** Agnieszka Wąsik, Irena Romańska, Lucyna Antkiewicz-Michaluk

**Affiliations:** Department of Neurochemistry, Institute of Pharmacology, Polish Academy of Sciences, Smętna 12, 31-343 Kraków, Poland

**Keywords:** Tetrahydroisoquinolines, Dopamine release, Microdialysis study, Rat striatum

## Abstract

The etiology of Parkinson’s disease (PD) may involve endogenous and exogenous factors. 1-Benzyl-1,2,3,4-tetrahydroisoquinoline (1BnTIQ), which was shown to be neurotoxic for dopaminergic neurons, is one of such factors, thus it can be used to construct an animal model of PD. In contrast, 1,2,3,4-tetrahydroisoquinoline (TIQ) and 1-methyl-1,2,3,4-tetrahydroisoquinoline (1MeTIQ) produce neuroprotective effects acting as monoamino oxidase (MAO) inhibitors and free radical scavengers that reduce oxidative stress in the mammalian brain. In this study, we aimed to investigate the effects of neuroprotective compounds, TIQ and 1MeTIQ, on the dopamine release in vivo in an animal model of PD induced by chronic administration of 1BnTIQ (25 mg/kg i.p.). Using an in vivo microdialysis methodology, we measured the impact of both acute and chronic treatment with TIQ and 1MeTIQ (50 mg/kg i.p.) on 1BnTIQ-induced changes in dopamine release in the rat striatum. Additionally, the behavioral test was carried out to check the influence of repeated administrations of the investigated compounds on the locomotor activity of rats. The behavioral studies showed that the chronic administration of 1BnTIQ produced a significant elevation of exploratory locomotor activity, and both the investigated amines, TIQ and 1MeTIQ, administered together with 1BnTIQ completely prevented 1BnTIQ-produced hyperactivity. The in vivo microdialysis studies demonstrated that the chronic treatment with 1BnTIQ caused a significant and long-lasting increase in the dopamine release (approximately 300 %) to the extracellular space in the rat striatum, which was demonstrated in the basal samples 24 h after 1BnTIQ injection. The combined chronic administration of 1BnTIQ and the investigated compounds, TIQ or 1MeTIQ, completely antagonized the 1BnTIQ-induced essential disturbances of the dopamine releasing to the extracellular space in the striatum. In conclusion, we suggest that higher concentrations of 1BnTIQ in the brain produced distinct impairment in the dopamine release, whereas TIQ and 1MeTIQ (compounds with previously revealed neuroprotective properties) completely prevented 1BnTIQ-induced abnormalities in the function of dopamine neurons and restored the dopamine release to the control values.

## Introduction

Tetrahydroisoquinolines are a group of endogenous compounds that are present in the mammalian brain (Abe et al. 2005; Yamakawa et al. 1999; Yamakawa and Ohta 1997, 1999). Some of these compounds, such as 1-benzyl-1,2,3,4-tetrahydroisoquinoline (1BnTIQ), have shown neurotoxic properties and are considered to be involved in the pathogenesis of Parkinson’s disease (PD) (Abe et al. 2001a; Kotake et al. 1995). Kotake et al. (1995) indicated that the concentration of 1BnTIQ in the cerebrospinal fluid (CSF) of parkinsonian patients was three times higher than that in the CSF of the control group. Previous studies revealed that chronic treatment with 1BnTIQ induces parkinsonian-like symptoms in mammals (Kotake et al. 1995, 2003, 2010). 1BnTIQ accumulates in dopaminergic neurons and can lead to parkinsonian symptoms. 1BnTIQ evoked strong activation of the oxidative MAO-dependent catabolic pathway (Wąsik et al. 2009). In addition, 1BnTIQ significantly inhibits the COMT-dependent O-methylation pathway. This mechanism of action leads to an increase in dopamine oxidation, and as a consequence, leads to an increase in reactive oxygen species (ROS) formation in dopaminergic neurons. Moreover, in vitro studies revealed that 1BnTIQ induces cell death via apoptosis and produces an increase in the formation of the active caspase-3 protein fragments (Shavali and Ebadi 2003). These data suggest that multiple administrations of 1BnTIQ might serve as an adequate animal model of the progressive process of PD. In contrast, 1,2,3,4-tetrahydroisoquinoline (TIQ), and especially its methyl derivative, 1-methyl-1,2,3,4-tetrahydroisoquinoline (1MeTIQ), have shown neuroprotective effects in the brain (Abe et al. 2001b; Antkiewicz-Michaluk et al. 2003, 2004, 2006; Wąsik et al. 2016). TIQ and 1MeTIQ are reversible MAO inhibitors that strongly block the MAO-dependent oxidative pathway and simultaneously increase the COMT-dependent O-methylation catabolic pathway. Therefore, both substances possess antioxidant properties. These compounds inhibit free radical formation and abolish H_2_O_2_ generation from dopamine via the Fenton reaction (Singer and Ramsay 1995; Antkiewicz-Michaluk et al. 2006; Patsenka and Antkiewicz-Michaluk 2004). Additionally, 1MeTIQ acts as a natural scavenger of free radicals. From a clinical point of view, the lack of a tolerance for its neuroprotective action after chronic treatment is both interesting and important (Antkiewicz-Michaluk et al. 2001; Wąsik et al. 2016).

To continue our previous studies, we would like to investigate the effects of TIQ and 1MeTIQ, as earlier demonstrated neuroprotective compounds, on the dopamine release in vivo in an animal model of PD induced by chronic administration of 1BnTIQ. Using in vivo microdialysis methodology, we measured the impact of acute and chronic treatment with TIQ and 1MeTIQ on 1BnTIQ-induced disorders of dopamine release in the rat striatum. In addition to the biochemical research, the behavioral test was carried out to check the influence of repeated administration of 1BnTIQ on the motor activity of rats.

## Materials and Methods

### Animals and Treatments

All experiments were carried out in male Wistar rats with an initial body weight of 280–300 g. All animals had free access to the standard laboratory food and tap water and were kept at room temperature (22°C) under an artificial light/dark cycle (12/12 h, light on at 7:00). The rats used for the microdialysis study after surgery were housed for several days individually. The experiments were carried out between 09:00 and 16:00 h. Control rats were treated with an appropriate solvent (0.9 % NaCl).

1BnTIQ was administered at a dose of 25 mg/kg intraperitoneally (i.p.) and chronically for 14 consecutive days. In the mixed group, TIQ or 1MeTIQ (50 mg/kg i.p.) was administered once 20 min before the last 1BnTIQ administration or was administered chronically 20 min before each 1BnTIQ injection.

All experimental procedures were performed in accordance with the National Institutes of Health Guide for the Care and Use of Laboratory Animals and were approved by the Bioethics Commission as compliant with the Polish Law. All experimental procedures were approved by the Local Bioethics Commission of the Institute of Pharmacology, Polish Academy of Sciences in Kraków.

### Drugs

1BnTIQ and 1MeTIQ were synthesized (according to Cannon and Webster 1958) at the Department of Drug Chemistry of the Institute of Pharmacology, the Polish Academy of Sciences in Krakow. The purity of the compounds was verified by measuring the melting point, and the homogeneity was assessed on a chromatographic column. TIQ (Sigma-Aldrich, USA) was obtained commercially. The compounds were dissolved in a 0.9 % NaCl solution.

### Behavioral Study

#### Locomotor Activity

Locomotor activity was assessed in actometers (Opto-Varimex activity monitors; Columbus Inst., USA) linked on-line to a compatible IBM PC. Each cage (43 × 44 × 25 cm) perimeter was lined with an array of 15 × 15 photocell beams located 3 cm from the floor. Interruptions of the photocell beams were counted as a measure of horizontal locomotor activity which was defined as the distance traveled (in cm). Rats were given 25 mg/kg 1BnTIQ i.p. as their last dose (14-day chronic treatment group). In the combined groups, TIQ or 1MeTIQ (50 mg/kg i.p.) was given chronically 20 min before each 1BnTIQ administration. Subsequently, the animals were transferred to experimental cages, and locomotor activity (horizontal activity, traveled distance in cm) was recorded for 90 min and analyzed using the Auto-Track Software Program (Columbus Instruments, USA). Each group comprised seven animals.

#### In Vivo Microdialysis

Rats were anesthetized with ketamine (75 mg/kg) and xylazine (10 mg/kg) and secured in a stereotaxic frame (Stoelting, USA). Vertical microdialysis guide cannulas (Intracerebral Guide Cannula with stylet; BAS Bioanalytical, USA) were implanted in the striatum (STR) according to the following stereotaxic coordinates: A/P +1.0, L/M +2.5, and V/D −3.5 mm from bregma and the dura (G. Paxinos and C.H. Watson). Fourteen days after surgery, microdialysis probes were inserted into the cannulas, and the striatum was perfused with an artificial cerebrospinal fluid (aCSF), which consisted of 140 mM NaCl, 2.7 mM KCl, 1.2 mM CaCl_2_, 1 mM MgCl_2,_ 0.3 mM NaH_2_PO_4_, and 1.7 mM Na_2_HPO_4_ (pH 7.4), at a flow rate of 1.5 μl/min with a microinfusion pump (Stoelting, Illinois USA). Samples were collected from freely moving rats in 20-min intervals after a 3-h wash-out period. 1BnTIQ was injected chronically (for 14 consecutive days) in a 25 mg/kg i.p., and dialysis samples were collected for 180 min. In the mixed groups, 1MeTIQ or TIQ (50 mg/kg i.p.) was administered 20 min before the last dose of 1BnTIQ (acute) or 20 min before each 1BnTIQ injection (chronic). All dialysates were immediately frozen on the dry ice (−70 ^ο^C), until they were used in a biochemical assay.

Levels of dopamine (DA) and its extraneuronal metabolite, 3-methoxytyramine (3-MT), were assayed in dialysates (20 μl) using HPLC with electrochemical detection as described below. The chromatograph Dionex Ultimate 3000 (Coulochem III, Germany) was equipped with C18 columns (Hypersil Gold; 150 mm × 3µ). The mobile phase comprised 0.05 M citrate–phosphate buffer (pH 3.5), 0.1 mM EDTA, 1 mM sodium octyl sulfonate, and 3.5 % methanol. The flow rate was maintained at 0.75 ml/min. Chromatographic data were processed using the Chromeleon Dionex computer programme (Germany). Dopamine and its metabolites were quantified by chromatograph peak height and compared with standards run on the day of the analysis. At the end of the experiment, frozen brains were examined histologically for correct probe placement. Each group consisted of six animals.

#### Calculations and Statistics

Data obtained from the locomotor activity and microdialysis studies were analyzed by a two-way analysis of variance (ANOVA) for repeated measures, followed by Duncan’s post hoc test, if significant differences were detected.

## Results

### The Effect of Chronic Administration of TIQ on 1BnTIQ-Induced Hyperactivity in Rats

**Fig. 1:**
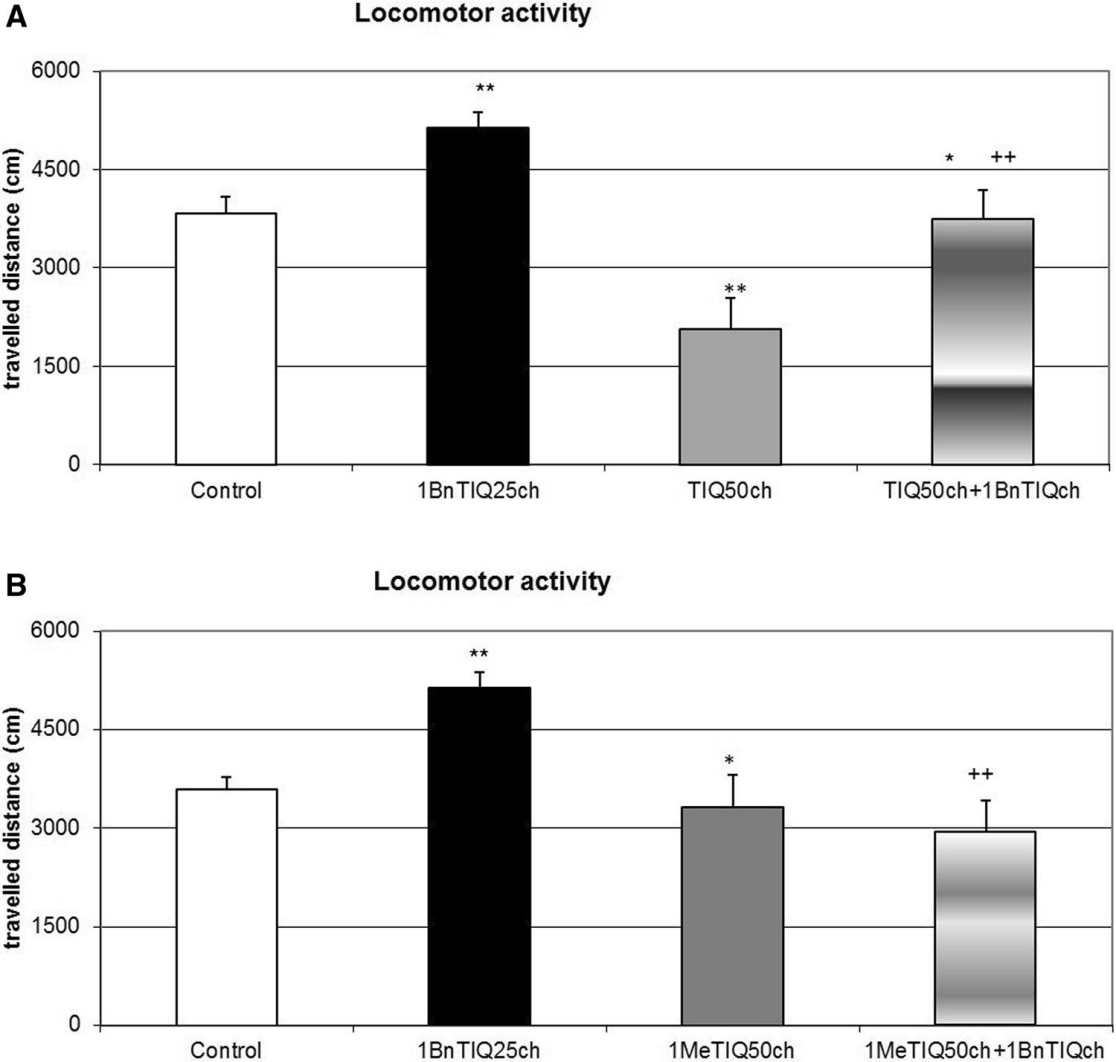
The effect of chronic administration of TIQ (**a**) or 1MeTIQ (**b**) on 1BnTIQ-induced hyperactivity in rats. Rats received a single injection of saline (control). 1BnTIQ was administered at a dose of 25 mg/kg i.p. chronically for 14 consecutive days. In the mixed group, TIQ (**a**) or 1MeTIQ (**b**) (50 mg/kg i.p.) was chronically administered 20 min before each 1BnTIQ injection. Rats were placed into actometers immediately after the treatment. Movements were recorded for 45 min. The data are expressed as the mean ± SEM (*n* = 6 animals). The data were analyzed with a two-way repeated measures ANOVA, followed by Duncan’s post hoc test. Statistical significance: *p < 0.05, **p < 0.01 versus saline-treated group; ^+^ *p* < 0.05, ^++^ *p* < 0.01 versus 1BnTIQ-treated group

The multiple 1BnTIQ (25 mg/kg i.p.) administrations produced a significant increase in the horizontal locomotor activity of rats (F[1,24] = 16.30; *p* < 0.01), while chronic administration of TIQ (50 mg/kg i.p.) induced a significant reduction in the locomotor activity of rats (F[1,24] = 18.29; *p* < 0.01). In the combined group, TIQ completely antagonized 1BnTIQ-induced hyperactivity (Fig. 1a).

### The Effect of Chronic 1MeTIQ Administration on 1BnTIQ-Induced Hyperactivity in Rats

As shown in Fig. 1b, the multiple (14-day) administrations of 1BnTIQ at a low dose of 25 mg/kg (i.p.) significantly increased (F[1,24] = 9.17; *p* < 0.01) the locomotor activity, while the chronic administration of 1MeTIQ (50 mg/kg i.p.) over 14 days induced a significant (F[1,24] = 10.65; *p* < 0.01) decrease in this parameter. In the joint 1BnTIQ + 1MeTIQ treatment group, rats showed the response similar to that for TIQ, i.e., 1MeTIQ completely antagonized the 1BnTIQ-induced hyperactivity (Fig. 1b).

### The Effect of Chronic 1BnTIQ Administration on the dopamine release in vivo

**Fig. 2:**
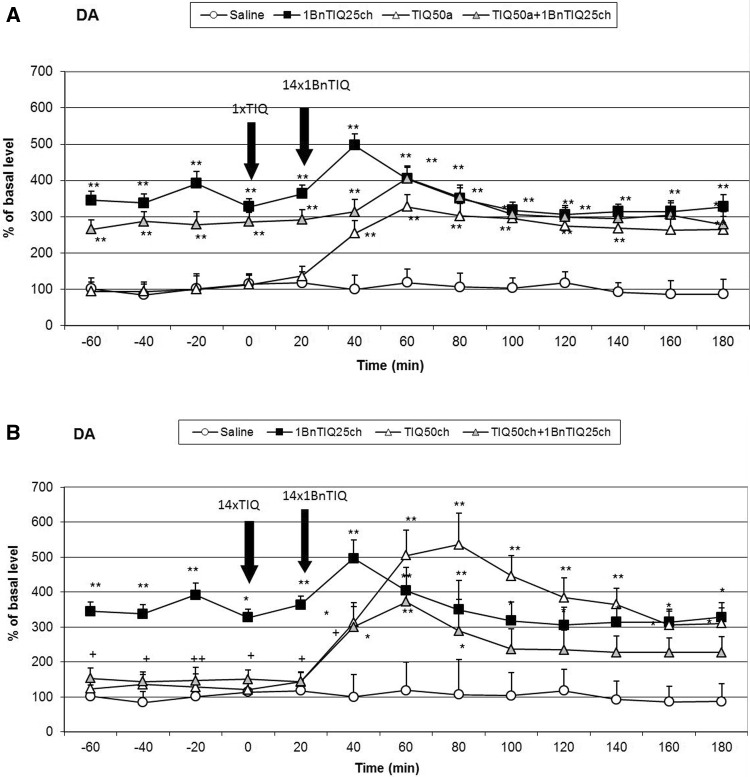
The effect of acute (**a**) or chronic (**b**) administration of TIQ on 1BnTIQ-induced changes on dopamine release in the rat striatum, 1BnTIQ was administered chronically (1BnTIQch) at a dose of 25 mg/kg i.p. for 14 consecutive days. In the mixed group, TIQ (50 mg/kg i.p.) was given acutely (TIQa) **a** 20 min before the last 1BnTIQ administration or chronically (TIQch), **b** 20 min before each 1BnTIQ administration. The control group was treated with saline. The dialysate was collected every 20 min. The concentration of dopamine (DA) was measured. The data are expressed as the mean ± SEM (*n* = 5–6). Statistical significance: *p < 0.05, **p < 0.01 from the basal value; ^+^ *p* < 0.05 versus 1BnTIQ-treated group (Duncan’s test)

The mean control basal extracellular concentration of dopamine in dialysates obtained from the striatum was approximately 17.8 ± 2.3 (pg/20 μl). A statistical analysis demonstrated a significant increase (aprox. 300 %; F[1,17] = 16.54; *p* < 0.01) in dopamine concentration in the synaptic cleft in the basal samples (from −60 to 0 min.) 24 h after chronic 1BnTIQ (25 mg/kg i.p.) administration. The last dose of 1BnTIQ fortified this effect approximately 200 %, and after 60 min, the concentration of dopamine returned to the value which was observed in the basal samples but it still was elevated about 300 % in comparison with the control group (saline) (Figs. 2a, b, 4a, b).

### The Effect of a Single or Chronic Administration of TIQ on 1BnTIQ-Induced Changes on the Dopamine Release in the Rat Striatum

After acute TIQ administration, an increase in the dopamine release was observed (approximately 300 %) (Fig. 2a). A post hoc analysis demonstrated that chronic 1BnTIQ administration, co-administered with acute TIQ, showed a significant increase in dopamine release in the striatum of approximately 300 % (F[1,16] = 18.18; *p* < 0.01) of the basal samples (Fig. 2a). However, after the last dose of 1BnTIQ and TIQ, the dopamine release increased up to 400 % within a short period (Fig. 2a).

In contrast to the effect of chronic 1BnTIQ, chronic administration of TIQ did not change the extracellular dopamine concentration estimated 24 h after the last injection (the basal samples; F[1,16] = 0.47; N.S.), but it increased the dopamine release after the last dose of TIQ (up to 500 %) (Fig. 2b). Interestingly, in the combined treatment group, chronic administration of TIQ completely blocked the effect induced by multiple administrations of 1BnTIQ (Fig. 2b). The dopamine concentration in the basal samples was similar to that in the control (saline group); however, after the last injection of TIQ and 1BnTIQ, the dopamine release was increased by approximately 300 % (F[1,16] = 15.38; *p* < 0.01) (Figs. 2b).

### The Effect of a Single or Chronic Administration of TIQ on 1BnTIQ-Induced Changes on the Concentration of 3-MT in the Rat Striatum

**Fig. 3:**
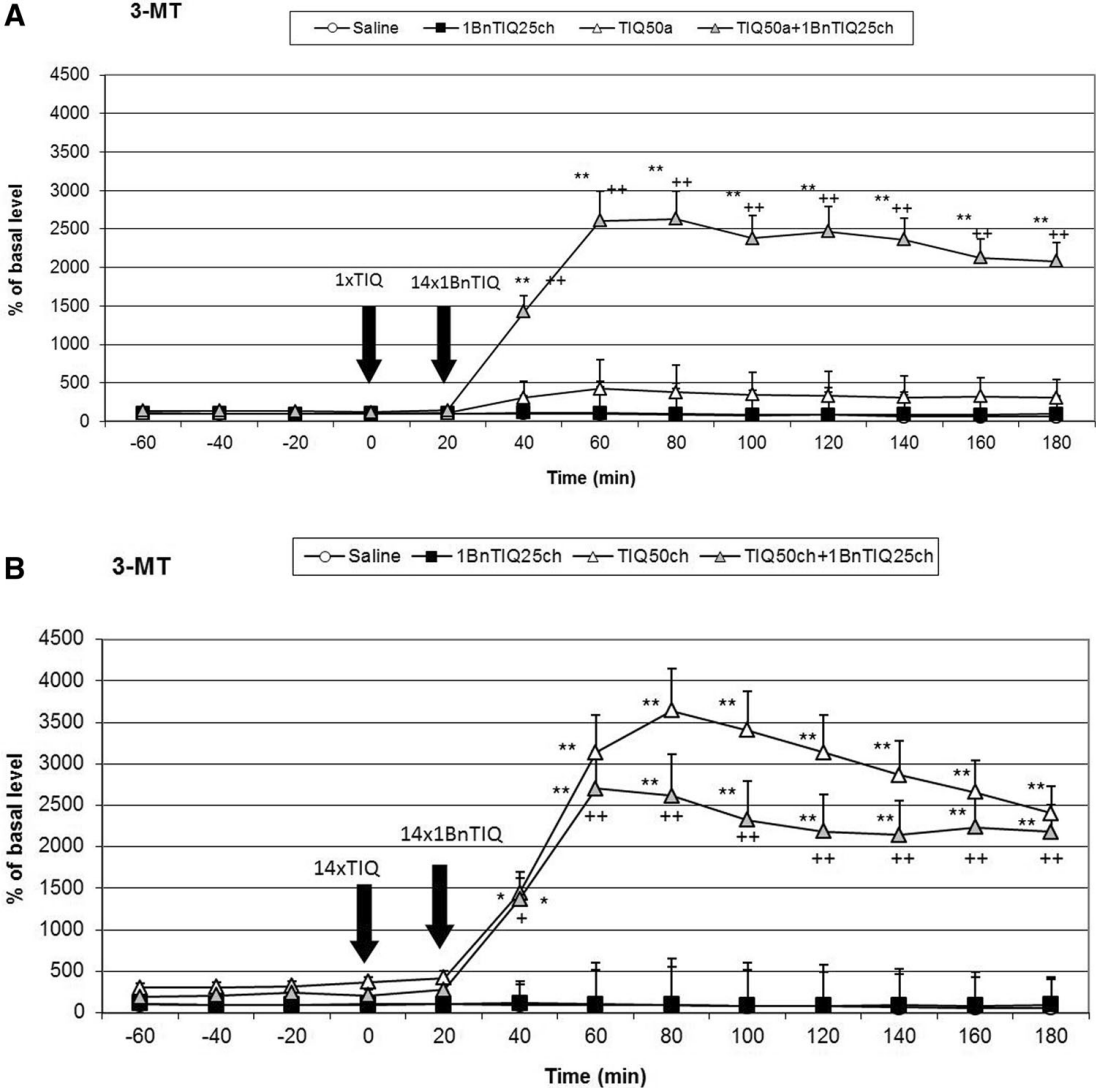
The effect of acute (**a**) or chronic (**b**) administration of TIQ on 1BnTIQ-induced changes in 3-MT concentration in the rat striatum, 1BnTIQ was administered chronically (1BnTIQch) at a dose of 25 mg/kg i.p. for 14 consecutive days. In the mixed group, TIQ (50 mg/kg i.p.) was given acutely (TIQa) **a** 20 min before the last 1BnTIQ administration or chronically (TIQch), **b** 20 min before each 1BnTIQ administration. The control group was treated with saline. The dialysate was collected every 20 min. The concentration of extraneuronal metabolite of dopamine 3-MT was measured. The data are expressed as the mean ± SEM (*n* = 5–6). Statistical significance: *p < 0.05, **p < 0.01 from the basal value; ^+^ *p* < 0.05 versus 1BnTIQ-treated group (Duncan’s test)

As shown in Fig. 3a, acute TIQ (50 mg/kg i.p.) administration produced a significant (F[1,16] = 19.14; *p* < 0.05) increase in the concentration of 3-MT in the rat striatum (up to 450 %). This effect was strongly potentiated in the combined treatment group (chronic 1BnTIQ + acute TIQ), (the concentration of 3-MT was elevated approximately 2500 %; F[1,16] = 11.84; *p* < 0.01) (Fig. 3a).

The post hoc analysis indicated that chronic TIQ (50 mg/kg i.p.) administration produced a huge and significant (F[1,16] = 37.61; *p* < 0.01) increase in the concentration of 3-MT in the rat striatum (up to 3500 %) (Fig. 3b). In the combined treatment group (with chronic treatment TIQ and 1BnTIQ), this effect was weaker, and the concentration of 3-MT was elevated up to 2500 % (F[1,16] = 30.94; *p* < 0.01) (Fig. 3b).

### The Effect of a Single or Chronic Administration of 1MeTIQ on 1BnTIQ-induced Changes in the Dopamine Release in the Rat Striatum

**Fig. 4:**
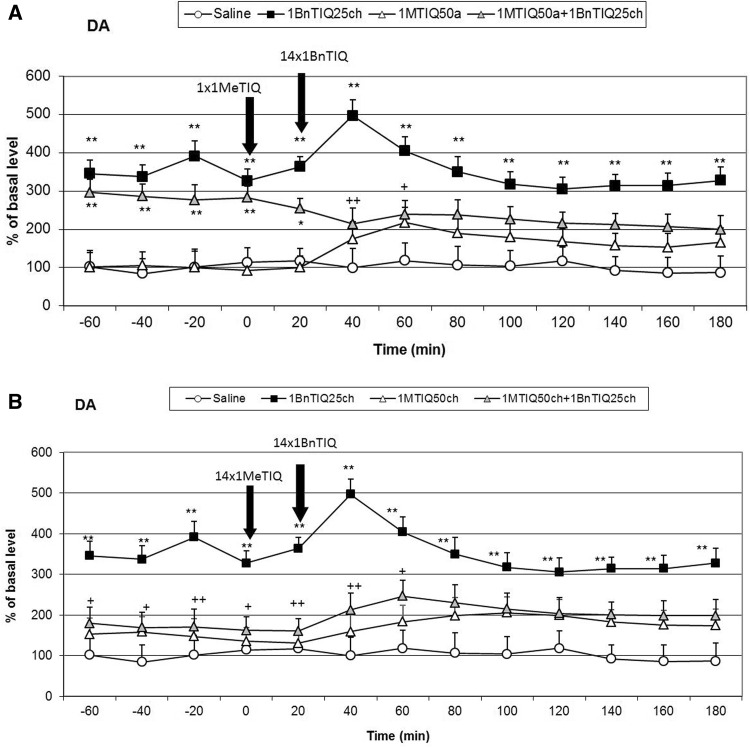
The effect of acute (**a**) or chronic (**b**) administration of 1MeTIQ on 1BnTIQ-induced changes in dopamine release in the rat striatum, 1BnTIQ was administered chronically (1BnTIQch) at a dose of 25 mg/kg i.p. for 14 consecutive days. In the mixed group, 1MeTIQ (50 mg/kg i.p.) was given acutely (1MeTIQa) **a** 20 min before the last 1BnTIQ administration or chronically (1MeTIQch), **b** 20 min before each 1BnTIQ administration. The control group was treated with saline. The dialysate was collected every 20 min. The concentration of dopamine (DA) was measured. The data are expressed as the mean ± SEM (*n* = 5–6). Statistical significance: *p < 0.05, **p < 0.01 from the basal value; ^+^ *p* < 0.05 versus 1BnTIQ-treated group (Duncan’s test)

An acute dose of 1MeTIQ produced an increase in the release of dopamine up to 200 % (Fig. 4a). Duncan’s test indicated that chronic 1BnTIQ + acute 1MeTIQ treatment induced a significant increase in the dopamine release in the rat striatum approximately 300 % (F[1,17] = 10.94; *p* < 0.01) of the basal samples (Fig. 4a). However, after the last dose of 1BnTIQ combined with an acute dose of 1MeTIQ, we observed a reduction in the release of dopamine to 200 % of the basal samples (Fig. 4a).

Chronic treatment with 1MeTIQ did not change the dopamine level in basal samples; however, a weak increase in dopamine release after the last dose of 1MeTIQ (up to 200 %; F[1,16] = 1.63; N.S.) was observed (Fig. 4b). Interestingly, in the combined treatment group, chronic injection of 1MeTIQ completely blocked the effect induced by repeated administrations of 1BnTIQ (Fig. 4b). The concentration of dopamine in basal samples was slightly above the control (saline) group, and after the final injection of 1MeTIQ and 1BnTIQ, the dopamine release increased by approximately 200 % (Fig. 4b).

### The Effect of Chronic 1BnTIQ Administration on 3-MT release in vivo

**Fig. 5:**
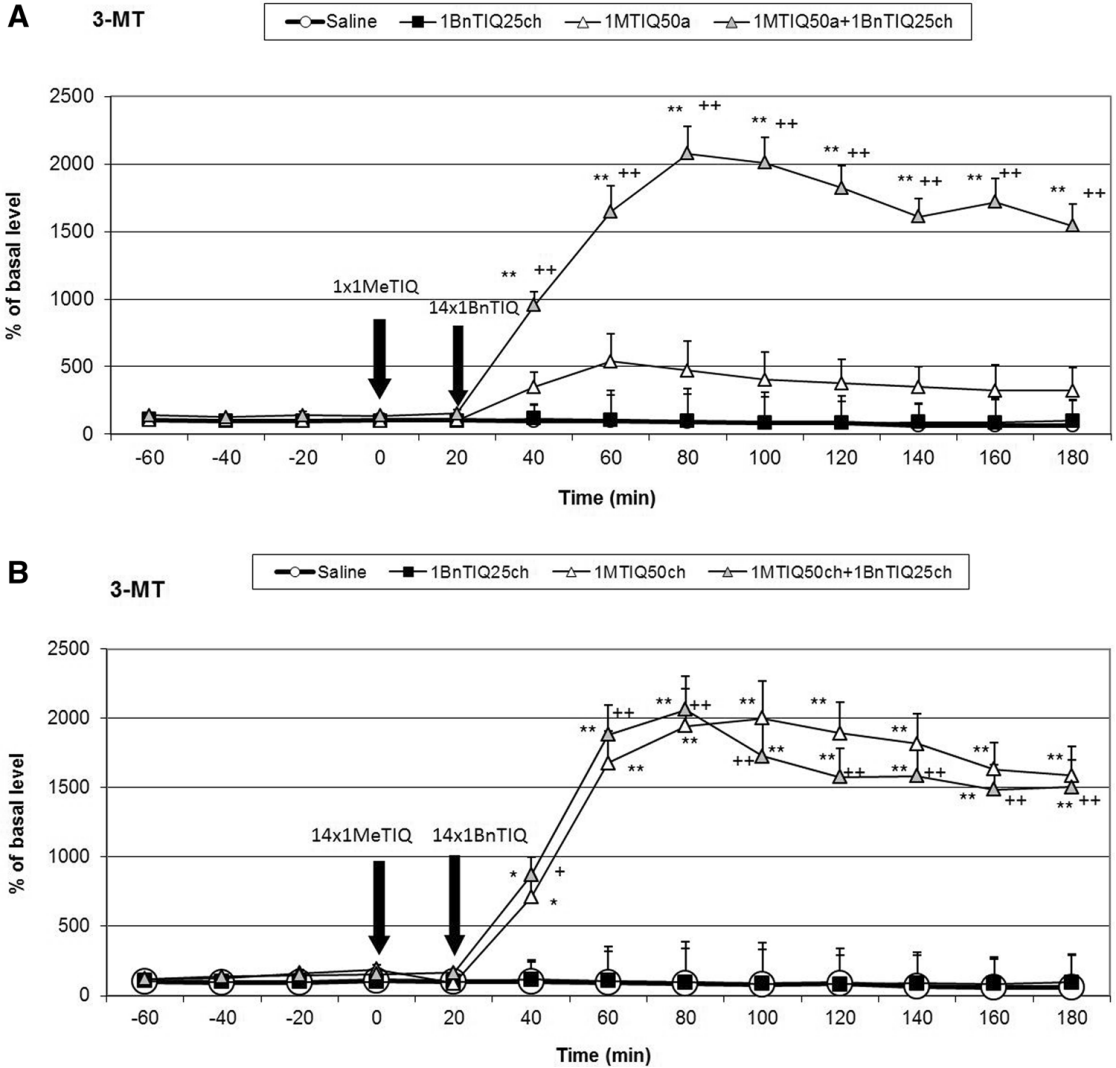
The effect of acute (**a**) or chronic (**b**) administration of 1MeTIQ on 1BnTIQ-induced changes in 3-MT concentration in the rat striatum, 1BnTIQ was administered chronically (1BnTIQch) at a dose 25 mg/kg i.p. for 14 consecutive days. In the mixed group, 1MeTIQ (50 mg/kg i.p.) was given acutely (1MeTIQa) **a** 20 min before last 1BnTIQ administration or chronically (1MeTIQch), **b** 20 min before each 1BnTIQ administration. The control group was treated with saline. The dialysate was collected every 20 min. The concentration of extraneuronal metabolite of dopamine 3-MT was measured. The data are expressed as the mean ± SEM (*n* = 5–6). Statistical significance: *p < 0.05, **p < 0.01 from the basal value; ^+^ *p* < 0.05 versus 1BnTIQ-treated group (Duncan’s test)

Duncan’s test indicated that chronic 1BnTIQ administration did not change the level of 3-MT (Figs. 3a, b, 5a, b).

### The Effect of a Single or Chronic Administration of 1MeTIQ on 1BnTIQ-Induced Changes on the Concentration of 3-MT in the Rat Striatum

As shown in Fig. 5a, acute 1MeTIQ (50 mg/kg i.p.) administration produced a significant (F[1,17] = 30.86; *p* < 0.05) increase in the concentration of 3-MT in the rat striatum (up to 500 %). This effect was strongly potentiated in the joint treatment group when 1MeTIQ was given with the last dose of 1BnTIQ (the concentration of 3-MT was elevated approximately 2000 %; F[1,17] = 14.03; *p* < 0.01) (Fig. 5a).

In contrast to the effect of chronic treatment with 1BnTIQ, multiple 1MeTIQ (50 mg/kg i.p.) administrations produced a huge and significant (F[1,17] = 52.41; *p* < 0.01) increase in the concentration of 3-MT in the rat striatum (up to 2000 %) (Fig. 5b). A similar effect was observed in the mixed group (with chronic treatment 1MeTIQ and 1BnTIQ), and the concentration of 3-MT was increased up to 2000 % (F[1,17] = 47.15; *p* < 0.01) (Fig. 5b).

## Discussion

The chronic administration of 1BnTIQ produced clear and significant disturbances in the function of dopaminergic neurons, which led to an increase in dopamine release in the striatum. Based on our previous experiments, we confirmed that chronic treatment with a low dose of 1BnTIQ (25 mg/kg i.p.) damaged the dopamine storage mechanisms causing its non-physiological rise in the extracellular space in the rat striatum (Wąsik et al. 2009, 2014). The main finding of the present study is that chronic administration of TIQ and 1MeTIQ completely prevented the disorders on dopamine release induced by multiple administrations of 1BnTIQ. Dopamine, the main neurotransmitter involved in motor control and Parkinson’s disease, is metabolized both intra- and extraneuronally. The extraneuronal dopamine metabolite, 3-MT, which is present in the synaptic cleft at relatively low concentrations comparable to dopamine, is considered to be a marker of dopamine release (Karoum et al. 1994). However, in contrast to intraneuronal dopamine metabolite, DOPAC and the final metabolite HVA, 3-MT may be biologically active. It was demonstrated previously that 3-MT has a considerable affinity as antagonist for noradrenergic α1 and dopamine D1 and D2 receptors in rat brain (Antkiewicz-Michaluk et al. 2008). In the behavioral tests chronic administration of 1BnTIQ-induced hyperactivity, while multiple injection of both, TIQ and 1MeTIQ significantly decreased the rats locomotor activity (Fig. 1a, b). Additionally, in the combined groups, TIQ and 1MeTIQ inhibited 1-BnTIQ-induced hyperactivity. In that light, the mechanism of the action of TIQ and 1MeTIQ, leading to an increase the concentration of 3-MT antagonism to 1BnTIQ-produced hyperactivity may be explained by its inhibition to catecholaminergic receptors.

Although all substances used in the present study belong to the same chemical group, their mechanisms of action are completely opposite. 1BnTIQ activates the dopamine oxidation pathway, which leads to the elevation in free radical production (Wąsik et al. 2014). On the other hand, TIQ and 1MeTIQ exhibit a contrasting molecular mechanism, and act as reversible MAO inhibitors, blocking the production of free radicals and activating the COMT-dependent O-methylation pathway (Antkiewicz-Michaluk et al. 2001; Wąsik et al. 2009). Our present in vivo study demonstrated that multiple administrations of 1BnTIQ produced a significant and long-lasting increase (approximately 300 % in comparison to the saline group) in dopamine release in the rat striatum, measured in the basal samples 24 h after its 13th injection (1 h before the last 14th injection). Such permanent increase in dopamine levels in the extracellular space after the chronic administrations of 1BnTIQ may, like the treatment with psychostimulants, leads to neurotoxic effects. After the 14th (last) dose of 1BnTIQ, dopamine release was elevated up to 500 % of the saline group; however, at the same time, no change in 3-MT concentration was observed. Moreover, 3-MT is considered as the most reliable indicator of dopamine release into the synaptic cleft (Egan et al. 1991; Karoum et al. 1994). Additionally, as demonstrated previously, 3-MT, in contrast to DOPAC and HVA, is an active metabolite of dopamine and possesses its own receptor activity (Antkiewicz-Michaluk et al. 2008; Alachkar et al. 2010). It is well known that elevation of dopamine release induces an increase of dopaminergic activity and leads to hyperactivity. The results of the in vivo microdialysis study are in agreement with the behavioral locomotor activity test in which chronic treatment with 1BnTIQ-produced hyperactivity in rats (Fig. 1a, b).

As we demonstrated earlier, 1BnTIQ similarly to reserpine (a specific vesicular monoamine transporter 2 (VMAT2) inhibitor) significantly depleted striatal dopamine (Wąsik et al. 2009). We postulated that 1BnTIQ might damage VMAT2 in dopaminergic neurons, leading to the pathological release of dopamine into the cytosol, and increased its MAO-dependent oxidation and free radical production (Wąsik et al. 2014). On the other hand, 1BnTIQ in low micromolar concentrations significantly inhibited the dopamine reuptake in slices of the rat striatum (Patsenka et al. 2004). Similarly, Okada et al. (1998) demonstrated that 1BnTIQ inhibited the uptake of [3H]dopamine by the dopamine transporter expressed in HEK293 cells. We suggest that DAT is responsible for the selective transport of 1BnTIQ into the dopaminergic neurons, leading to the 1BnTIQ neurotoxicity correlated with impaired dopamine storage via inhibition of VMAT2 (Wąsik et al. 2009). Since TIQ and 1MeTIQ have also affinity to DAT, both substances may restrict access of 1BnTIQ to the DAT (Patsenka et al. 2004). This mechanism results in a reduction of penetration of 1BnTIQ into the neurons. The present study revealed that acute treatment with TIQ induced an increase in dopamine release (approximately 300 %), while 1MeTIQ showed a similar effect of a lesser magnitude (Figs. 2a, 4a). Taking into account that both investigated compounds, TIQ and 1MeTIQ, act as reversible MAO inhibitors, they shift dopamine catabolism toward the COMT-dependent O-methylation pathway, which leads to a significant increase in the extraneuronal concentration of the dopamine metabolite 3-MT (Antkiewicz-Michaluk et al. 2001; Patsenka and Antkiewicz-Michaluk 2004). In the present paper, such properties of TIQ and 1MeTIQ were confirmed in our in vivo microdialysis studies which demonstrated an increase in 3-MT level by approximately 500 % in the case of acute administration and up to 2500 % versus saline group after chronic treatment. It is worth emphasizing, as previously demonstrated that 3-MT, an extraneuronal dopamine metabolite, shows affinity for α1-adrenergic and D1 and D2 receptors as an antagonist, and may play an important role as an inhibitory regulator, counteracting the excessive stimulation of catecholaminergic neurons (Antkiewicz-Michaluk et al. 2008; Alachkar et al. 2010). Thus, a high concentration of 3-MT may play an important role in locomotor activity as dopamine D2 and noradrenergic α1 receptors antagonists (Antkiewicz-Michaluk et al. 2008). Such mechanism of action of 3-MT leads to the paradoxical effect, where despite the increase in dopamine release, we observed decrease in the locomotor activity in rats after treatment with both, TIQ and 1MeTIQ (Fig. 1a, b).

In the combined treatment group, an acute dose of TIQ given before the last dose of 1BnTIQ only slightly reduced the effect of 1BnTIQ, whereas acute administration of 1MeTIQ produced a clear decrease in dopamine release (Figs. 2a, 4a). In both combined treatment groups, a massive increase in 3-MT levels was observed (2500 and 2000 %, respectively; Figs. 3a, 5a). Such an increase in the 3-MT concentration may be associated with the retention of dopamine in the extracellular space, due to dopamine reuptake blockade by TIQ and 1MeTIQ, and catabolism of all available dopamine by the COMT-dependent O-methylation pathway. It is worth emphasizing that such a mechanism of action for TIQ and 1MeTIQ inhibits the production of free radicals, which are formed during the catabolism of dopamine by MAO-dependent oxidation, and consequently leads to a reduction in oxidative stress (Antkiewicz-Michaluk et al. 2006; Miller et al. 1996; Patsenka and Antkiewicz-Michaluk 2004).

Interestingly, in both combined treatment groups, chronic treatment with TIQ or 1MeTIQ completely antagonized the 1BnTIQ-induced pathological increase in dopamine release in the basal samples (Figs. 2b, 4b). Simultaneously, in these groups, we observed a huge elevation in the concentration of 3-MT after the last dose of TIQ and 1MeTIQ (up to 2500 and 2000 %, respectively) (Figs. 3b, 5b). These results are important because they demonstrate that chronic administration of both TIQ and 1MeTIQ can counteract the disturbances in dopamine release produced by multiple administrations of 1BnTIQ. We suggest that TIQ and 1MeTIQ given before each 1BnTIQ injection inhibit DAT and in this way block penetration of 1BnTIQ into the neurons. Since 1BnTIQ penetrates into cells via DAT, DAT is inhibited by prior injection of TIQ and 1MeTIQ; therefore, in the combined treatment groups, we did not observe the effect of 1BnTIQ (Patsenka et al. 2004). The data from the behavioral tests (locomotor activity) confirmed that a high concentration of 3-MT, which was observed in both joint treatment groups, acted as an endogenous neuroleptic, as previously demonstrated (Antkiewicz-Michaluk et al. 2008), and could block hyperactivity in rats (Fig. 1a, b). It is important to mention that pronounced increases in dopamine O-methylation in the COMT-dependent pathway may provide neuroprotection (Antkiewicz-Michaluk et al. 2001; Miller et al. 1996). It was also indicated that elevation of 3-MT in the process of dopamine O-methylation protected cells against oxidative stress (Miller et al. 1996). The neuroprotective properties of 1MeTIQ against different dopaminergic neurotoxins, including MPTP, 6-OHDA, rotenone, and 1BnTIQ, were also demonstrated in in vitro studies in cultured mesencephalic neurons (Kotake et al. 2005) and in ex vivo studies (Antkiewicz-Michaluk et al. 2011; Wąsik et al. 2016).

## Conclusions

Summing up the results, based on the in vivo microdialysis study presented in this paper, we suggest that both endogenous amines TIQ and 1MeTIQ can antagonize 1BnTIQ-induced clear disorders of dopamine release in vivo in rat striatum. Furthermore, these amines were able to restore the physiological functions of striatal dopamine neurons disturbed by chronic application of 1BnTIQ.
